# Distributed Fault Detection Based on Credibility and Cooperation for WSNs in Smart Grids

**DOI:** 10.3390/s17050983

**Published:** 2017-04-28

**Authors:** Sujie Shao, Shaoyong Guo, Xuesong Qiu

**Affiliations:** State Key Laboratory of Networking and Switching Technology, Beijing University of Posts and Telecommunications, Beijing 100876, China; syguo@bupt.edu.cn (S.G.); xsqiu@bupt.edu.cn (X.Q.)

**Keywords:** distributed fault detection, diagnosis request, suspicious sensor, credibility model, neighbor cooperation

## Abstract

Due to the increasingly important role in monitoring and data collection that sensors play, accurate and timely fault detection is a key issue for wireless sensor networks (WSNs) in smart grids. This paper presents a novel distributed fault detection mechanism for WSNs based on credibility and cooperation. Firstly, a reasonable credibility model of a sensor is established to identify any suspicious status of the sensor according to its own temporal data correlation. Based on the credibility model, the suspicious sensor is then chosen to launch fault diagnosis requests. Secondly, the sending time of fault diagnosis request is discussed to avoid the transmission overhead brought about by unnecessary diagnosis requests and improve the efficiency of fault detection based on neighbor cooperation. The diagnosis reply of a neighbor sensor is analyzed according to its own status. Finally, to further improve the accuracy of fault detection, the diagnosis results of neighbors are divided into several classifications to judge the fault status of the sensors which launch the fault diagnosis requests. Simulation results show that this novel mechanism can achieve high fault detection ratio with a small number of fault diagnoses and low data congestion probability.

## 1. Introduction

With the rapid development and deployment of smart grids, the number and types of power and communication infrastructures are gradually increasing and the networks are becoming more complex. For scientific decision-making and efficient operation management, a large number of sensors are deployed to accurately and comprehensively monitor infrastructures and network status information, such as voltage, current, temperature, humidity, frequency, and so on [1,2]. Due to the complicated and harsh working environment of these sensors in smart grids, such as vibration, noise, channel interference, fire and ice disaster, and so on, perceiving data incorrectly or permanent sensor faults occur commonly, which will severely affect the connectivity and real-time monitoring capability of the supporting wireless sensor networks (WSNs) [3]. Therefore, accurate and timely fault detection is the key issue that needs to be solved for WSNs in smart grids.

WSN fault management was preliminarily summarized and some research directions discussed in [4]. After lots of research by relevant scholars, there are two main methods of sensor fault detection nowadays: centralized mode and distributed mode. The centralized mode usually detects sensor faults by periodically collecting measurement and status information of sensors at a central node, which requires more complicated computations of the global information data and results in fast energy consumption of sensors near the central node due to the large amount of data communication and this shortens the lifetime of the WSN [5,6,7]. It is very expensive to identify faulty sensors in a centralized mode. The distributed mode usually adopts a method whereby each sensor compares its own monitoring data with the data of neighboring sensors to detect its potential faults [8,9]. This is a good way to overcome the problem of unbalanced energy consumption among sensors. Moreover, a low implementation complexity and high probability of correct fault diagnosis can be achieved. Thus, a distributed fault detection mechanism is highly preferred for WSNs in smart grids.

For distributed fault detection, there is no need to periodically launch information communication among all of sensors for detecting potential faults, but only to exchange information between some sensors and their neighbors. Statistical techniques are usually adopted to identify the sensors that are suspected to be faulty [8,9,10,11]. Besides the sensing data of neighbors, the sensor’s own historical data are also used. However, these methods usually consider that most of sensors have a probability to be faulty in a certain area, and launch the fault detection process for each sensor. This still brings pressure on the limited computation and energy resources of the WSN, and there is room for improvement in the fault detection efficiency. Therefore, distinguishing suspicious sensors from healthy sensors first may be a potential choice when starting a fault detection process in a WSN. The credibility and the confidence level of sensors are introduced into the fault detection algorithm, and the identification of suspicious sensors is started as the first step in fault detection [12]. Then, fault detection begins with the chosen sensors which are suspected of being faulty according to the data exceptions. Some data exceptions that occur due to some actual unexpected event that happens are identified and no fault detection will be launched. For example, the monitoring data of a temperature sensor will dramatically change when the temperature of its monitoring area soars [13]. Therefore, it is necessary to judge whether the sensor data is truly suspicious.

Moreover, there may be a large number of suspicious sensors in the same time period, and some of them are neighbors of each other. If all of them launch fault diagnosis requests at the same time this would probably lead to data congestion, mutual interference, and further energy consumption of the sensors [14,15]. Unordered and repeated data communications between sensors should be avoided. Thus, another problem of distributed fault detection is the decision-making of fault diagnosis requests, which contains two parts: sending sensor and sending timing of fault diagnosis requests. Besides, to guarantee the accuracy of fault diagnosis results, classification statistics of the diagnosis replies from neighbors are discussed [16].

Based on the above analysis, this paper presents a distributed fault detection mechanism based on credibility and cooperation for WSNs in smart grids. Firstly, the credibility model of a sensor is established to judge the suspicious status of sensors according to their own temporal data correlation. Based on the credibility model, a suspicious sensor is chosen to launch fault diagnosis requests. Secondly, the sending time of fault diagnosis request is discussed to avoid the transmission overhead brought by unnecessary diagnosis requests and improve the efficiency of fault detection based on neighbor cooperation. The diagnosis replies of neighboring sensors are analyzed according to their own status. Finally, to further improve the accuracy of fault detection, the diagnosis results of neighbors are divided into several classifications to judge the fault status of sensors.

The rest of this paper is organized as follows: in Section 2, related work is introduced. In Section 3, a sensor credibility model is established. A distributed fault detection mechanism based on credibility and cooperation is proposed and discussed in Section 4. Simulation results are analyzed in Section 5. Section 6 draws the conclusions of this work.

## 2. Related Work

Nowadays, the volume of literature on studies about distributed fault detection for WSNs is increasing rapidly. A large number of works have looked at the efficiency and accuracy of fault detection methods in the different WSN application fields. Most research indicates that neighbor cooperation is the basic idea of distributed fault detection methods [12,13,14,15,16,17], but the point at issue in the research is the specific way of cooperation, such as which sensor the fault detection process should begin with, timing of fault diagnosis requests, and so on.

A fault detection mechanism based on historical data and neighbor cooperation is proposed in [17], which selects the healthy sensor with the largest number of neighbors as the root node and iteratively judges the status of its neighbors. It indicates that fault detection only based on data of single healthy sensor is not enough to get a reliable result. In [18,19], the probability of faults is calculated based on the data of neighboring sensors, and the probability is adjusted by the data of the boundary sensors. These methods judge potential faulty sensors through the corresponding probability, but the calculation of probability through data difference needs to be optimized. Reference [20] proposes a fault recognition algorithm based on distributed hash table, which achieves the fault diagnosis by comparing with data of neighbor sensors. A *k*-means based fault detection algorithm is proposed in literature [21], and an optimized ant colony algorithm is used to improve the accuracy of fault detection to a certain extent. However, the high complexity of the algorithm limits its wide application in large-scale WSN, especially in smart grid.

Launching diagnosis requests to the neighbor sensors immediately once the status of some sensors becomes suspicious is proved to be an unreasonable choice [12,16]. Although these methods compare the current data of suspicious sensors with their own historical data and data of their neighbors, respectively, the unordered and repeated transmission communications of fault diagnosis requests have a great impact on the efficiency and accuracy of fault detection. Reference [22] discusses the transmission timing of fault diagnosis requests and adopts the time window mechanism to determine the appropriate timing for sending diagnosis requests, but the execution mode is not specific.

All of above studies assume that most of the neighboring sensors of the suspicious sensors are healthy. However, in practical implementations, error perception data and sensor faults probably have a spatial correlation. The specific status of neighbor sensors should be considered during fault detection of the suspicious ones to further improve the accuracy. It is obvious that the classification of neighboring sensors by status is an effective way to improve the judging efficiency and accuracy of fault diagnosis requests. Therefore, we can further design a similar approach to analyze the fault detection for WSNs in smart grids.

## 3. Credibility Model of a Single Sensor

**Figure 1:**
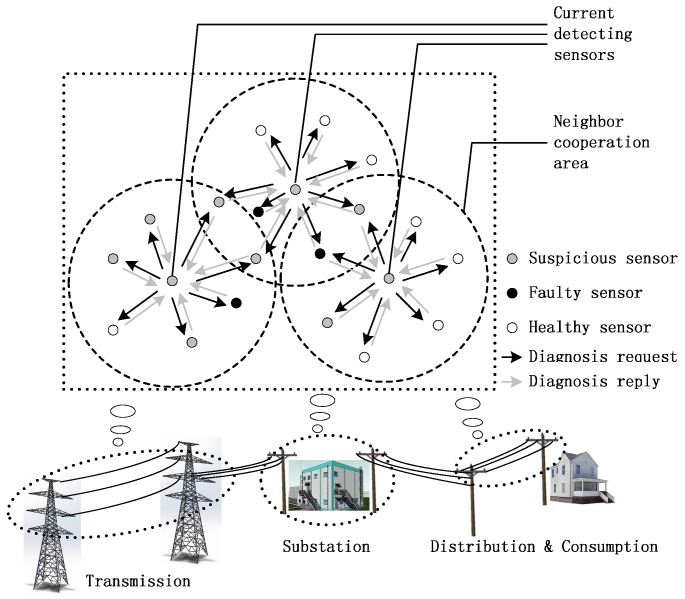
Distributed fault detection for WSNs in smart grids.

Distributed fault detection based on credibility and cooperation for WSNs in smart grids is shown in Figure 1. Most sensors are mainly deployed to monitor power system infrastructure such as power transmission towers, substations, poles, and so on. They are used to collect the status information, such as voltage, current, temperature, humidity, frequency, and so on, thus there may be various types of sensors in a smart grid WSN.

In this paper, those different types of sensors are just considered as ones with same functions during the fault detection process and it is assumed they can store their own data and communicate with each other. Based on this assumption, we begin to discuss the fault detection mechanism.

Because of their data storage function, single sensors can judge whether its own status is suspicious or not according to the temporal correlation of data when there is a data exception. When the data exception is judged as indeed caused by some actually happened unexpected event, the sensor does not need to launch the fault detection process. Therefore, a reasonable and accurate credibility model of a single sensor should be established firstly.

We assume that a single sensor can store *k* data in its limited memory. At time *t*, those *k* data are $d_{1},d_{2},\cdots ,d_{k}$ at time t−k,t−k+1,⋯,t−1, respectively. We denote $E_{t-1}$ as the mean value of $d_{1},d_{2},\cdots ,d_{k}$, $E_{t-1}=\sum_{i=1}^{k}d_{i}/k$. Then, the variance $S_{t-1}^{2}$ of $d_{1},d_{2},\cdots ,d_{k}$ is calculated as:

(1) $$S_{t-1}^{2}=\sum_{i=1}^{k}(d_{i}-E_{t-1}{)}^{2}/k$$

We denote $d_{t}$ as the data at time *t*. Then, the mean value $E_{t}$ of the current k data is calculated as $E_{t}=\left(d_{2}+d_{3}+\cdots +d_{k}+d_{t}\right)/k$. Then, the variance $S_{t}^{2}$ is calculated as:

(2) $$S_{t}^{2}=\frac{\sum_{i=2}^{k}(d_{i}-E_{t}{)}^{2}+{\left(d_{t}-E_{t}\right)}^{2}}{k}$$

We set a positive threshold value *s*. If $\left|S_{t}^{2}-S_{t-1}^{2}\right|<s$, the gap of variances of two adjacent times is small, which represents that the current data is consistent with the historical data [16]. If $\left|S_{t}^{2}-S_{t-1}^{2}\right|\geq s$, then the current data generates an exception.

We denote θ,(0≤θ≤1) as the credibility of each sensor to judge whether the data exception is caused by an actually happened unexpected event or not. The initial value of θ is set as 1. We set a positive value ∂, (0<∂<1). If $\left|S_{t}^{2}-S_{t-1}^{2}\right|\geq s$, θ reduces by ∂, otherwise θ increases by ∂. A series of continuous data with close values or the same values may result in θ increasing to a value that is larger than 1. Because the data with close values or same values make the variance change a little or even not change at all, and this makes the value of θ increase. Thus, we set the condition that the value of θ must not be larger than 1. θ would converge as the data gradually updates.

However, adjusting the credibility only depending on the variance would ignore some accumulated sensor faults, such as so-called stuck-at-N and stuck-at-zero sensor faults. Assuming that one sensor fails at time *t* and its monitoring data abnormally increases, its following data may keep being large like the data at time *t*. The value of $\left|S_{t}^{2}-S_{t-1}^{2}\right|$ may gradually decrease or even be less than *s* over time, and the value of the credibility θ may increase instead, which results in this fault not being able to be monitored. However, the value of $\left|E_{t}-E_{t-1}\right|$ may increase and maintain a relatively large value over time in this case, which can be used to monitor this fault. Therefore, we set another positive threshold value *e* about the mean value to adjust the credibility θ with the threshold *s* together. If $\left|S_{t}^{2}-S_{t-1}^{2}\right|<s$ and $\left|E_{t}-E_{t-1}\right|<e$, then θ increases, otherwise θ decreases. The credibility model is established using Equation (3):

(3) $$\theta =\left\{ \begin{array}{l} 1,\;\left|S_{t}^{2}-S_{t-1}^{2}\right|<s,\left|E_{t}-E_{t-1}\right|<e,\theta +\partial \geq 1\\ \theta +\partial ,\left|S_{t}^{2}-S_{t-1}^{2}\right|<s,\left|E_{t}-E_{t-1}\right|<e,\theta +\partial <1\\ \theta -\partial ,else \end{array}\right.$$

**Theorem** **1.** *The values of e and s are determined by the differences between any two reasonable data* $d_{1}$*,* $d_{t}$ *and* $E_{t-1}$*, and have nothing to do with the specific values of* $d_{1}$*,* $d_{t}$ *and* $E_{t-1}$*.*

**Proof.**  $E_{t}-E_{t-1}=\left(d_{t}-d_{1}\right)/k$, according to Equation (3), $\left|E_{t}-E_{t-1}\right|<e$. Then, $\left|\left(d_{t}-d_{1}\right)/k\right|<e$. *k* is constant. Thus, the value of e is determined by difference between $d_{1}$ and $d_{t}$.

Then $E_{t}=E_{t-1}+(d_{t}-d_{1})/k$, according to Equation (2):

$$S_{t}^{2}=\frac{\sum_{i=2}^{k}{\left(d_{i}-E_{t-1}-\left(d_{t}-d_{1}\right)/k\right)}^{2}}{k}+\frac{{\left(d_{t}-E_{t-1}-\left(d_{t}-d_{1}\right)/k\right)}^{2}}{k}$$

$$kS_{t}^{2}=\sum_{i=2}^{k}{\left(d_{i}-E_{t-1}\right)}^{2}-\frac{2(d_{t}-d_{1})\sum_{i=2}^{k}\left(d_{i}-E_{t-1}\right)}{k}+\frac{\left(k-1\right){\left(d_{t}-d_{1}\right)}^{2}}{k^{2}}+{\left(d_{t}-E_{t-1}-\left(d_{t}-d_{1}\right)/k\right)}^{2}$$

According to Equation (1), then

$$kS_{t}^{2}=kS_{t-1}^{2}-{\left(d_{1}-E_{t-1}\right)}^{2}+{\left(d_{t}-E_{t-1}\right)}^{2}-\frac{{\left(d_{t}-d_{1}\right)}^{2}}{k}$$

$$S_{t}^{2}-S_{t-1}^{2}=\frac{k{\left(d_{t}-E_{t-1}\right)}^{2}-k{\left(d_{1}-E_{t-1}\right)}^{2}\;-{\left(d_{t}-d_{1}\right)}^{2}}{k^{2}}$$

According to Equation (3), $\left|S_{t}^{2}-S_{t-1}^{2}\right|<s$, then

$$\frac{\left|k{\left(d_{t}-E_{t-1}\right)}^{2}-k{\left(d_{1}-E_{t-1}\right)}^{2}\;-{\left(d_{t}-d_{1}\right)}^{2}\right|}{k^{2}}<s$$

Thus, the value of *s* is determined by the differences between any two reasonable data of $d_{1}$, $d_{t}$ and $E_{t-1}$, and has nothing to do with the specific values of $d_{1}$, $d_{t}$ and $E_{t-1}$. ☐

According to Equation (3), we set a threshold ε as the dividing point between credibility of suspicious sensor and healthy sensor, as shown in Equation (4).

(4) $$\left\{ \begin{array}{l} \theta >\varepsilon ,\;healthy\;sensor\\ \theta \leq \varepsilon ,\;suspicious\;sensor \end{array}\right.$$

**Theorem** **2.** *To make sure the status of sensor is turned to suspicious when there are continuous m data exceptions* (0<m<k,m∈Ζ)*, the values* ∂ *and* ε *should meet the demand of* 1−m∂≤ε<1−(m−1)∂*.*

**Proof**. According to Equation (3), θ is reduced by ∂ for each data exception. When there are continuous m data exceptions, θ≤1−m∂. According to Equation (4), if the sensor is turned into a suspicious status, then 1−m∂≤ε. For a further accurate value of ε, ε<1−(m−1)∂. Thus, if 1−m∂≤ε<1−(m−1)∂, then the status of the sensor is certainly turned into suspicious when there are continuous m data exceptions. ☐

Of course, a sensor can be turned into a suspicious status without *m* continuous data exceptions when ε<θ<1. For example, if θ=1−(m−1)∂ at time t−2, then θ=1−(m−2)∂ with normal data at time t−1. θ=1−m∂, if the data are normal at time t and t+1. Then, the sensor is suspicious. This situation usually occurs when the data fluctuates between abnormal and normal status multiple times, in which case it is necessary to judge that the sensor is really suspicious.

The value of *m* in Theorem 2 is set according to the actual requirements. m=1 means that fault detection is launched immediately once a data exception happens. A larger value of *m* can reduce the network data transmission pressure, but may ignore some transient faults. A smaller value of *m* can improve the accuracy of fault detection, but may cause a lot of unnecessary data transmission overhead. Therefore, there is a trade-off between the accuracy of fault detection and the network traffic volume to determine the optimal value of *m*.

Although the credibility model can detect most faults, it may ignore one type of stuck-at-N sensor fault. If the sensor value is stuck to the last correct measurement, and the values of historical data change smoothly or they are similar to the stuck one or even the same, then this kind of fault can’t be detected by our proposed credibility model. To solve the problem we set a clock timer for each healthy sensor. When the time exceeds the threshold, which means the sensor has never experienced a round of fault detection since the clock timer is reset, the credibility of the sensor is set as suspicious and diagnosis requests are sent to perform fault detection. Then, this fault can be detected.

## 4. Fault Detection Mechanism Based on Credibility and Cooperation

In this section, the fault detection mechanism based on credibility and cooperation (FD-CAC) is proposed. Based on the credibility model, a sensor can effectively capture data exceptions which can be suspected to be caused by faults. Next, the suspicious sensor needs to be tested to discover if it is indeed in faulty status or not. In this paper, the fault detection is based on neighbor cooperation and the notion of the spatial correlation of data. As is shown in Figure 1, the basic fault detection procedure for a single sensor is that the suspicious sensor sends fault diagnosis requests to its neighboring sensors which are in a circular area with itself at the center and *R* as the radius, and these neighboring sensors send fault diagnosis replies back to make the suspicious sensor judge its own status. In this paper, we a mainly concerned with fault-free sensors, i.e., sensors that can still communicate and process when their sensing modules are faulty. To guarantee that our proposed FD-CAC actually works, we assume that the communication modules of all suspicious sensors operate well.

### 4.1. Diagnosis Request

#### 4.1.1. Diagnosis Request Parameter

Because there may be several types of sensors in the real monitoring area in a smart grid, the data measurement scales or data dimensions among neighbor sensors may be different. The effect of data measurement scale or data dimensions should be eliminated to avoid inappropriate comparisons. We adopt the coefficient of variation as the first parameter of a diagnosis request instead of variance.

We denote $CV_{t}$ as the data coefficient of variation of the k data at time *t*. $CV_{t}$ is calculated by:

(5) $$CV_{t}=\sqrt{S_{t}}/E_{t}$$

There is a geographical distance between each of the various sensors which are scattered in the monitoring area. In order to accurately evaluate the impact of the geographical factor on the data variety, we assign the coordinate *L* of the suspicious sensor as one of the parameters of a diagnosis request.

In addition, in order to identify the diagnosis request and avoid the potential information loss caused by a large number of burst data communications when multiple suspicious sensors in the same area simultaneously launch diagnosis requests, we assign the time *t* as the third parameter of diagnosis request. Therefore, the diagnosis request sent by sensor $N_{i}$ is denoted as $DREQ(i,CV_{t},L,t)$.

#### 4.1.2. Diagnosis Request Timing

When multiple sensors send fault diagnosis requests at the same time, data conflict and information loss may occur and some diagnosis requests may be sent another time. To improve the efficiency and alleviate the potential data congestion, the diagnosis request timing should be well analyzed and planned.

Generally, the effective way of distinguishing these fault diagnosis requests is dispersing them on the time axis. In this paper, we adopt an equal-probability time window mechanism to determine the fault diagnosis request timing. All suspicious sensors in the cooperative area adopt the same time window. We denote *l* and tn as the length of one time interval and the number of time intervals in the time window, respectively. The values of *l* and tn are set according to the real-time monitoring requirements of equipment and other specific circumstances. The probability that each suspicious sensor sends a diagnosis request to its neighbors in each time window [t+i×l,t+(i+1)×l], i=0,1,...,tn−1 is equal. Assuming that there are *n* sensors that turn into suspicious ones at time *t*, the probability that these sensors send diagnosis requests at the same time window is $1/tn^{n-1}$. The average expected number of diagnosis requests that sensors send in each time window is n/tn, which is reduced by tn times.

When the time window of the suspicious sensor $N_{i}$ arrives, $N_{i}$ sends a diagnostic request $DREQ(i,CV_{t},L,t)$ to its neighbor sensors. Thus, the diagnosis requests of these *n* suspicious sensors are greatly dispersed and the data transmission pressure is effectively alleviated.

### 4.2. Diagnosis Reply Based on Neighbor Cooperation

After receiving a diagnosis request $DREQ(i,CV_{t},L,t)$ sent by a suspicious sensor $N_{i}$, its neighbor sensor $N_{j}$ which is in the circular area with $N_{i}$ as the center and *R* as the radius would send its diagnosis reply back to $N_{i}$. 

We denote $CV_{t}^{0}$ as the data coefficient of variation of $N_{j}$ at time *t*. We denote $DCV_{t}$ as the difference between $CV_{t}$ and $CV_{t}^{0}$, $DCV_{t}=CV_{t}-CV_{t}^{0}$. $N_{j}$ calculates the distance $dis_{ij}$ between the two sensors and deals with the diagnosis request according to its own different status.

If $N_{j}$ is healthy, its diagnosis reply should be calculated with Equation (6), which indicates that a sensor whose data variation is similar with that of healthy sensor may obviously be healthy unless it breaks a certain data boundary. λ is set according to the actual circumstances, which represent the spatial correlation of monitoring data of sensors.

(6) $$rep=\left\{ \begin{array}{l} \frac{\left|DCV_{t}\right|}{dis_{ij}},\;\frac{\left|DCV_{t}\right|}{dis_{ij}}<\lambda \\ -\frac{\left|DCV_{t}\right|}{dis_{ij}},\;\frac{\left|DCV_{t}\right|}{dis_{ij}}\geq \lambda \end{array}\right.$$

If $N_{j}$ is faulty, its diagnosis reply should be calculated using Equation (7), which indicates that a sensor whose data variation is similar to that of faulty sensor has a high probability of being faulty, otherwise it may be healthy. The diagnosis replies from faulty sensors provide the fault judgement from the perspective of faulty data. They do not violate the actual data correlations among a suspicious sensor and its neighboring sensors. They can be used as supplements of the diagnosis replies from healthy sensors, and can be used to relatively reduce the data processing error of diagnosis replies from healthy sensors to improve the fault detection ratio and efficiency, especially in the cases where most neighbor sensors in the neighboring cooperation area are faulty ones:

(7) $$rep=\left\{ \begin{array}{l} -\frac{\left|DCV_{t}\right|}{dis_{ij}},\;\frac{\left|DCV_{t}\right|}{dis_{ij}}<\lambda \\ \frac{\left|DCV_{t}\right|}{dis_{ij}},\;\frac{\left|DCV_{t}\right|}{dis_{ij}}\geq \lambda \end{array}\right.$$

There are two main types of faults of fault-free sensors. The faulty sensor $N_{j}$ can be divided into one of two types: sensors with sensing faults and sensor with communication faults. The first one is the sensor whose data sensing module is faulty, but its communication module operates well. Although there may be data exceptions, it can send back diagnosis replies. The other one is the sensor whose communication module is faulty, so it can’t send diagnosis replies back. Here, we mainly focus on the diagnosis replies of the first type of sensors. The method of fault detection of the second type of sensors will be discussed in the next subsection.

If sensor $N_{j}$ is suspicious, it can’t judge the status of $N_{i}$ according to its own status. To some extent, its data is not informative enough to perform the fault detection. Therefore, its diagnosis reply should be calculated as:

(8) rep=0

Then, $N_{j}$ sends its fault diagnosis reply message DREP(j,rep,status,t) back to $N_{i}$.status∈{1,0,−1} represents $N_{j}$ is in a healthy, suspicious or faulty status, respectively.

### 4.3. Fault Judgement Based on Modified Credibility

We denote *T* as the time period taken for sending data from $N_{i}$ to the sensor at the edge of the neighbor cooperation area. Obviously, the time periods taken by sending data from $N_{i}$ to most other sensors in the neighbor cooperation area are less than *T*. We set that $N_{i}$ will wait for *2T* after sending diagnosis requests for each round of fault judgement. With the 2*T* waiting time, there is time left for $N_{i}$ to finish the computation of most of the replies although each computation is not complicated, and the unification and simplification of the process of fault judgement are obtained. During the *2T* waiting time, the diagnosis replies are received and dealt with the method of classification statistics as follows:

We denote $n_{h}$, $n_{s}$, $n_{f}$ as the number of diagnosis replies sent by the healthy, suspicious, and faulty neighboring sensors, respectively. They are calculated using Equation (9):

(9) $$\left\{ \begin{array}{l} n_{h}=n_{h}+1\;if\;status=1\\ n_{s}=n_{s}+1\;if\;status=0\\ n_{f}=n_{f}+1\;if\;status=-1 \end{array}\right.$$

We denote sn as the total number of diagnosis replies that received by $N_{i}$ during the *2T* waiting time, then $sn=n_{h}+n_{f}+n_{s}$. Assuming all sn DREP.rep are $rep_{1},rep_{2},...,rep_{sn}$, then we calculate the diagnosis result of this time fault detection based on neighbor cooperation as:

(10) $$Trep=\sum_{i=1}^{sn}rep_{i}$$

To effectively and directly evaluate the impact of this diagnosis result, we transform the diagnosis result into the change ratio of credibility $\theta_{r}$:

(11) $$\theta_{r}=\frac{Trep}{\sum_{i=1}^{sn}\left|rep_{i}\right|}$$

Then, we modify the credibility of sensor $N_{i}$ using Equation (11):

(12) $$\theta =\theta \times (1+\theta_{r})$$

Next, in order to improve the accuracy of fault detection, we judge the status of $N_{i}$ based on the modified credibility by classifications. The possible classifications are as follows:

(a) $n_{h}>\frac{sn}{2}$

More than half of the neighbor sensors of $N_{i}$ are healthy. There are few faulty sensors and suspicious sensors. The status of $N_{i}$ is judged as follows:

If θ>ε, according to the Equation (1), $N_{i}$ can be directly judged as a healthy sensor. However, if θ≤ε, $N_{i}$ should not be simply judged as a faulty sensor based for the purpose of further accuracy of faulty detection. In this case, if $\theta_{r}>0$, which means the most healthy neighboring sensors consider the data variance of $N_{i}$ is relatively similar to their own, although it is not enough, so we launch another round of the fault diagnosis process to get further accurate detection results. Thus, only if $\theta \leq \varepsilon \;and\;\theta_{r}\leq 0$, $N_{i}$ should be directly judged as a faulty sensor. Another round of fault diagnosis process with *2T* waiting time should be launched if $\theta \leq \varepsilon \;and\;\theta_{r}>0$.

(b) $n_{f}>\frac{sn}{2}$

More than half of the neighboring sensors of $N_{i}$ are faulty. There are few healthy sensors and suspicious sensors. The status of $N_{i}$ is judged as follows:

Obviously, if θ>ε, $N_{i}$ can be directly judged as a healthy sensor. If θ≤ε, $N_{i}$ can be directly judged as a faulty sensor too. In this case, most diagnosis replies are calculated with Equation (7), and most of them are reverse deduced with the probability method. Moreover, the probability of a fault is high for a sensor whose neighboring sensors are mostly faulty according to the actual smart grid application environment. Therefore, we directly judge $N_{i}$ as a faulty sensor if θ≤ε in this classification.

(c) $n_{h}+n_{f}>\frac{sn}{2}\;and\;n_{h}<\frac{sn}{2}\;and\;n_{f}<\frac{sn}{2}$.

The number of faulty neighboring sensors and the number of healthy neighboring sensors are all less than a half, but the sum of the two is more than half. Obviously, if θ>ε, $N_{i}$ can be directly judged as a healthy sensor as in the previous two classifications. However, if θ≤ε, the status of $N_{i}$ is judged for two cases:

(1) $n_{h}\geq n_{f}$
  The healthy sensors are more than the faulty ones. For the similar reason of classification (a) in this section, another round of fault diagnosis process with *2T* waiting time should be launched if $\theta \leq \varepsilon \;and\;\theta_{r}>0$, and $N_{i}$ should be directly judged as a faulty sensor if $\theta \leq \varepsilon \;and\;\theta_{r}\leq 0$.
(2) $n_{h}<n_{f}$
  In this case, the faulty sensors are more than the healthy ones. For the similar reason given in classification (b), we directly judge $N_{i}$ as a faulty sensor if θ≤ε.

(d) $n_{h}+n_{f}<\frac{sn}{2}$

There are lots of suspicious neighbor sensors, and obviously $n_{s}>n_{h}\;and\;n_{s}>n_{f}$. In this case, the diagnosis replies are not referential. It should wait another *2T* to allow some suspicious neighboring sensors judge their own status and launch another fault diagnosis process round.

After the process of faulty judgement based on modified credibility, we modify the credibility of the suspicious sensor and identify its status according to the judgement. The credibility of a sensor with healthy status is set to 1, and that with a faulty status is set to 0. Finally, the fault information is sent to the operation management system in the control center to get immediate and accurate fault recovery.

In the previous subsection, we mentioned another type of fault where the communication module of sensor is faulty. This faulty sensor can’t respond to any diagnosis requests. Thus, a further solution is needed. Obviously, $N_{i}$ can’t receive the diagnosis replies from this type of sensors during the *2T* waiting time. We denote $sn_{0}$ as the total number of diagnosis requests that are sent by $N_{i}$. Then, we get the number of this type of sensors, which is expressed as $sn_{0}-sn$. We set a positive integer threshold value α. To put it simply, we consider that there are enough diagnosis replies for $N_{i}$ to finish fault judgement if $sn_{0}-sn<\alpha$, and we continue operate our proposed FD-CAC. Otherwise, we consider that most of the sensors are suspected to be faulty in the neighboring cooperation area of $N_{i}$, and the credibility of $N_{i}$ is set to 0. Then the high-level fault information is sent to the operation management system in the control center to ask for a comprehensive check of the sensors in this area, and finally get immediate and accurate fault recovery.

Moreover, we have to focus on another abnormal problem. The sensors may be compromised and they can cheat on the credibility since they calculate the credibility themselves. Cheating on the credibility may result in two cases. The first one is that the data are disguised as abnormal ones. The other one is that the data are disguised as normal ones. Obviously, the sensor will be suspected in the first case, and this fault can be detected by our proposed FD-CAC. In the second case, the credibility of the sensor keeps indicating a healthy status, and this fault may be ignored. To solve this problem, we set a clock timer for each healthy sensor. When the time exceeds the threshold, which means the sensor has never experienced a round of fault detection since the clock timer was reset, the credibility of the sensor is set as suspicious and diagnosis requests are sent to initiate the fault detection process. Then this fault can be detected. The clock timer is reset when the sensor is judged as a healthy one again.

## 5. Simulation Results

In this section, the performance of our proposed FD-CAC fault detection mechanism is evaluated through MATLAB numerical simulations. We mainly simulate the instantaneous and permanent faults of sensing modules, and the fault of communication modules. To accurately simulate the realistic faults of sensors in a smart grid environment, we adopt the random fault model with area relevance, which means that the faults randomly happen and some of the same type of faults may happen together with high probability in a geographically close area. Because some sensor faults happen due to the drastic changes of the external environment rather than slow abrasion of internal components and the cascade characteristics of smart grid will have an impact on the fault type and lead to the occurrence of cascaded faults [23,24,25]. The specific fault models are as follows: stuck-at fault, offset fault and random noise fault. In the design of our FD-CAC, the value of *m* is the factor that will greatly affect the performance of fault detection. Firstly, 30, 60, and 90 sensors are randomly scattered in 100 × 100 m^2^ area, and simulations are carried out under these three sensor densities to determine the optimal value of *m*. Secondly, to further evaluate the fault detection performance, we compare FD-CAC with the weighted average-based fault detection algorithm (FD-WA) proposed in [9], the neighbor-data analysis-based fault detection schema (FD-NDA) proposed in [12] and the throughput descent and energy efficient mechanism for fault detection (FD-TDEE) proposed in [16] because all of them make fault judgements based on neighbor comparisons, and both of FD-NDA and FD-TDEE adopt the method that a sensor can start fault detection process whenever it finds its reading data is suspicious by comparison between new sensing data and historical data. To ensure statistical validity, the data used in the simulation results analysis are averaged and all simulation experiments are repeated 100 times.

**Figure 2:**
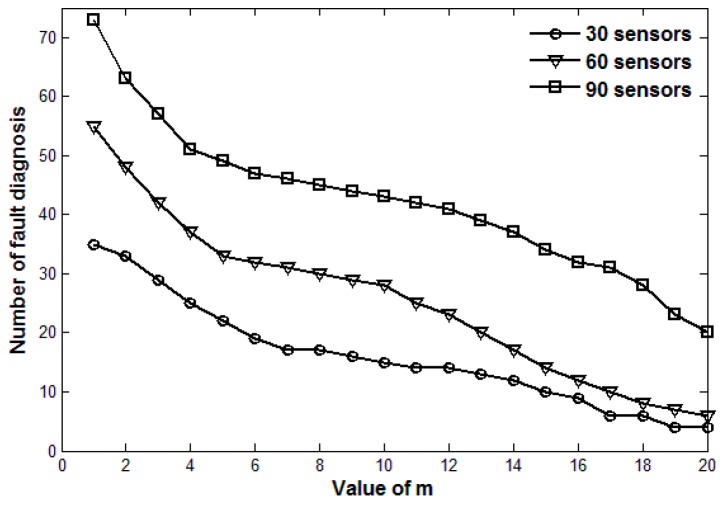
Number of fault diagnosis with the change of *m* for three sensor densities.

**Figure 3:**
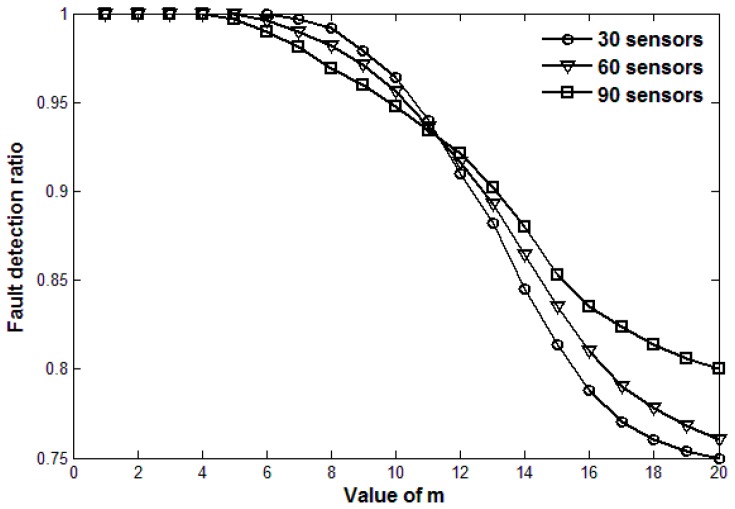
Fault detection ratio with the change of *m* for three sensor densities.

Figure 2 and Figure 3 show the number of fault diagnoses and fault detection ratio, respectively, with the increasing value of *m* and three different sensor densities. Figure 2 shows that the number of fault diagnosis decreases as the value of *m* increases. The smaller the value of *m* is, the fewer the number of continuous data exceptions is required for fault diagnosis, which usually results in a larger number of fault diagnoses. Figure 3 shows that the fault detection ratio decreases as the value of *m* increases. The larger the value of *m* is, the larger number of data exceptions required for fault diagnosis is, which may lead faults with fewer continuous data exceptions to be ignored. However, a large number of fault diagnoses would result in great data communication pressure. Thus, there is a trade-off between the number of fault diagnoses and the fault detection ratio when we choose a reasonable value of *m*. As is shown in Figure 2 and Figure 3, when the values of *m* are respectively 6, 5 and 4 for 30, 60 and 90 sensors, the fault detection ratio is 100% and the number of fault diagnosis is relatively acceptable. When the values of *m* are respectively 11, 11 and 10, the number of fault diagnoses is much fewer and fault detection ratio still remains about 95%.

**Figure 4:**
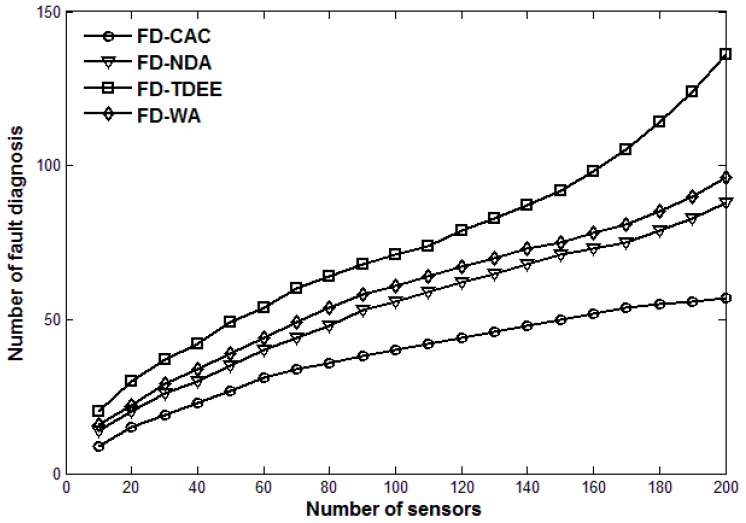
Number of fault diagnosis for four fault detection mechanisms.

**Figure 5:**
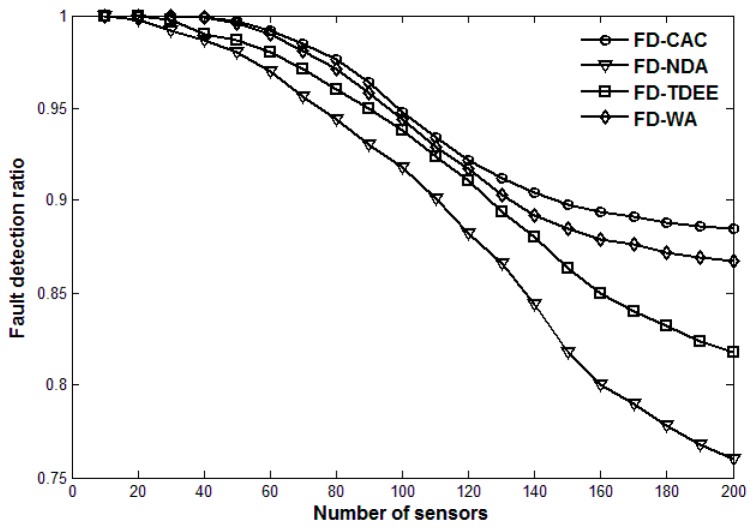
Fault detection ratio for four fault detection mechanisms.

Next, we compare the performance of FD-CAC with those of FD-WA, FD-NDA and FD-TDEE. Figure 4 and Figure 5 show the number of fault diagnoses and fault detection ratio of the four fault detection mechanisms (*m* = 10), respectively. As shown in Figure 4, the number of fault diagnoses of FD-CAC is the least of the four, and that of FD-TDEE is the most. Although both the FD-NDA and FD-TDEE mention that the suspicious status of a sensor should be judged first, their methods are defective. FD-TDEE only adopts variance of historical data to calculate the credibility of a sensor. If the continuous data exceptions are maintained at a relatively small range of fault data, the credibility of a sensor may gradually increase, in which case a potential fault may be ignored. FD-NDA adopts a mean value of historical data to calculate the credibility of a sensor. When large and small data exceptions occur simultaneously, the data exception may not be captured due to the average of the two abnormal data values. Moreover, judgement only depending on mean value will generate some unnecessary suspicious sensors because of the consistency of actual data changes. FD-WA adopts an exchange of data between a sensor and all its neighbors to calculate a weighted-average, which needs some rounds to get an accurate result. In this paper, FD-CAC establishes the credibility model of a sensor by combining both the variance and mean value, which can avoid missing the data exceptions mentioned above and reduce the unnecessary fault diagnosis process. When the credibility of a sensor is lower than a certain threshold, the fault diagnosis request would be sent, which can be effectively proved to reduce unnecessary data communication overhead.

Figure 5 shows that the fault detection ratio of FD-CAC is the best of the four, and that of FD-NDA is the worst. In addition, the fault detection ratio of FD-CAC changes only a little as the number of sensor increases, but those of FD-NDA and FD-TDEE decrease rapidly. After receiving the diagnosis replies from the neighbor sensors, FD-NDA judges the status of a suspicious sensor as a healthy one only depending on whether more than half of the neighbor sensors consider the suspicious one as healthy, otherwise it is deemed faulty. This method does not take into account the status of neighboring sensors because the replies of the suspicious neighboring sensors are not informative. FD-WA also ignores this, but its weighted-average and confidence level make its result relatively accurate. FD-TDEE mentions the classification judgement, but it ignores the classification where most neighboring sensors are faulty. Thus, our proposed FD-CAC judges faults through four main classifications according to the status statistics of neighboring sensors, which can be effectively proved to improve the fault detection ratio and maintain a relatively high level as the number of sensors in the monitoring area increases.

In this paper, it is assumed that the sensors are scattered in a regular area. Actually, the monitoring area of a WSN is irregular in smart grids, specifically in situations in the wild. However, the efficiency and speed of fault detection are figured out, and the simulation results show that our FD-CAC fault detection mechanism can be used as a reference in large-scale WSNs in smart grids.

## 6. Conclusions

This paper presents a novel distributed fault detection mechanism based on credibility and cooperation (FD-CAC) for WSNs in smart grids. Firstly, we establish a reasonable credibility model of a single sensor according to its own temporal data correlation. The suspicious status of a sensor is identified according to its credibility level. Then the suspicious sensor will launch fault diagnosis requests to its neighboring sensors. We discuss the optimum sending time of fault diagnosis requests to avoid the transmission overhead caused by unnecessary diagnosis requests and improve the efficiency of fault detection based on neighbor cooperation. We analyze the diagnosis replies of neighboring sensors according to their own status. Finally, to further improve the accuracy of fault detection, the diagnosis replies of neighbors are divided into four groups to judge the fault status of a sensor. Simulation results show that this novel mechanism can achieve high fault detection ratios with a few fault diagnosis times and low data congestion probability, and especially it can maintain relatively high performance in a large-scale WSN in a smart grid.

As a future task, we will seek to further study the feasibility of a reasonable diagnosis reply of suspicious sensors and the corresponding fault judgement method when the neighboring sensors of the chosen one are almost suspicious in order to improve the speed and efficiency of fault detection to effectively guarantee the monitoring work of WSNs in smart grids.
